# Headpulse Biometric Measures Following Concussion in Young Adult Athletes

**DOI:** 10.1001/jamanetworkopen.2023.28633

**Published:** 2023-08-11

**Authors:** Cathra Halabi, Lynda Norton, Kevin Norton, Wade S. Smith

**Affiliations:** 1 Department of Neurology, University of California, San Francisco; 2 Weill Institute for Neurosciences, University of California, San Francisco; 3 Alliance for Research in Exercise, Nutrition and Activity (ARENA), Allied Health and Human Performance, University of South Australia, Adelaide, Australia

## Abstract

**Importance:**

Concussions are common in sports. Return-to-play protocols can be enhanced by objective biometrics.

**Objective:**

To characterize temporal changes of headpulse, a digital biometric, in athletes with sports-related concussion; to explore the association of unstructured physical activity with headpulse changes.

**Design, Setting, and Participants:**

This cohort study included headpulse measurements from players in the highest level of amateur Australian Rules Football in South Australia. Analysis included feasibility and validation phases, with the feasibility cohort recruited between August 5, 2021, and September 10, 2021, and the validation cohort recruited between May 5, 2022, and September 3, 2022. Data were analyzed October 2022 through January 2023.

**Interventions:**

Cranial accelerometry detected micromovements of the head following cardiac contraction (what we have described as “headpulse”). Headpulse was serially recorded for 1 month in concussed individuals.

**Main Outcomes and Measures:**

Headpulse waveforms underwent frequency transformation analysis per prespecified algorithm. Result *Z* scores were calculated. Headpulse *Z* scores exceeding 2 (2 SDs from control means) met an abnormality threshold. Headpulse sensitivity, timing, and duration of change were determined.

**Results:**

A total of 59 control and 43 concussed individuals (44 total concussions; 1 control also concussed, 1 concussed individual injured twice) provided headpulse measurements. The feasibility cohort (all male) included 17 control (median [IQR] age, 23 [19-28] years) and 15 concussed individuals (median [IQR] age, 21 [19-23] years). The validation cohort included 25 female (median [IQR] age, 21 [20-22] years) and 17 male (median [IQR] age, 26 [23-29] years) control individuals, and 8 female (median [IQR] age, 28 [20-31] years) and 20 male (median [IQR] age, 21 [19-23] years) concussed individuals. Headpulse reached abnormality threshold in 26 of 32 concussed individuals (81%; 9% on day 0, 50% by day 2, 90% by day 14). Headpulse alterations lasted 14 days longer than symptoms and were exacerbated by return-to-play or unsupervised physical activity.

**Conclusions and Relevance:**

In this study of 101 amateur Australian Rules Football athletes, the digital headpulse biometric was evaluated in 44 sports-related concussions. Compared with controls, new headpulse changes occurred after concussion; this objective metric may complement return-to-play protocols.

## Key Points

**Question:**

Are there temporal changes in cranial accelerometer-derived headpulse biometric values after sports-related concussions?

**Findings:**

In this cohort study of 43 concussed and 59 control athletes with 44 total concussions, 26 of 32 concussed individuals (81%) were identified by headpulse biometric analysis. A more pronounced biometric change was found in multiple participants after return-to-play despite symptom resolution.

**Meaning:**

These results suggest serial measurement of the headpulse biometric reveal characteristic changes after concussions and additional alterations occur in athletes returning to play within 1 month; the headpulse biometric warrants further study and has potential to complement return-to-play decisions.

## Teaser

This cohort study of high-level amateur Australian Rules Football players measures and validates headpulse, a novel biometric measure for assessing sports-related concussions.

## Introduction

Traumatic brain injury (TBI) causes significant morbidity and mortality with an annual global incidence exceeding 60 million.^1^ One third of injuries are sports-related with predilection for young adults.^2^ Concussion, including sports-related concussion (SRC), is a subset of mild TBI. Symptoms include headache, vestibular impairment, visual changes, cognitive symptoms, mood changes, and sleep disturbance.^3,4^ Another concussion during recovery can be neurologically deleterious and in rare cases fatal (“second impact syndrome”).^5^ To mitigate reinjury risk, most organized sports teams use concussion protocols that determine return-to-play (RTP). Objective measures of concussive injury and definitive recovery are an unmet need in SRC.^6^

We present a digital biomarker derived from cranial accelerometry in this study. Cranial accelerometry draws on principles of ballistocardiography first recognized in the 19th century.^7^ While ballistocardiography measures whole body forces produced by cardiac contraction,^7,8,9^ our study focuses on measuring cardiac output forces directed toward the head. Highly sensitive accelerometers within our headset noninvasively measure cardiac forces sustained by the head. We call the resulting waveform the “headpulse.” We previously showed that frequency domain analysis of headpulse supports diagnosis of concussion and recovery.^10^ We have also shown headpulse abnormalities in moderate to severe TBI,^11^ large vessel stroke,^12,13^ cerebral vasospasm,^14^ and cardiac arrest.^15^ In this study, we evaluated a longitudinal cohort of concussed and control athletes of amateur Australian Rules Football to investigate the time course of headpulse signal change (Australian and University of California San Francisco Concussion Study in Athletes [AUSSIE-1], referred to throughout as A1). Our model was then validated in a second cohort (AUSSIE-2, A2). In Australian Rules Football,^16^ opposing unhelmeted teams score by moving a ball toward goalposts at either end of a large field. This involves running, kicking, or punching the ball. Tackling or jumping on an opponent are common maneuvers. It is a distinct contact and collision sport originating from Australia with some similarities to rugby, soccer, and US football, and there are international amateur leagues cultivating the sport more broadly.^17,18^ Recovery protocol includes 24 to 48 hours of strict rest, followed by graded individual then team training, provided there is no symptom exacerbation; the earliest allowed RTP after protocol completion and medical clearance is 12 days after a concussion.^19,20^

## Methods

This cohort study involved athletes from the Adelaide Football League, the highest level of amateur Australian Football in Adelaide, South Australia. The study was approved by the Human Research Ethics Committee of Bellberry Limited, a national, private not-for-profit organization which provides scientific and ethical review of human research studies in Australia.^21^ The study was conducted following the Strengthening the Reporting of Observational Studies in Epidemiology (STROBE) reporting guideline for observational studies. It was performed in 2 phases to confirm feasibility and refine methodology (A1) then validate findings (A2); A2 additionally explored the association of physical activity with headpulse patterns and included female athletes.

### Participants

A total of 762 athletes across 9 clubs provided written consent at season start. Active athlete controls were recruited from 1 club and were concussion free in the preceding year. Concussed individuals were recruited across all clubs as de novo concussion occurred. The A1 cohort (male athletes) was recruited between August 5, 2021, and September 10, 2021. The A2 cohort (male and female athletes) was recruited between May 5, 2022, and September 3, 2022.

### Procedures

The study was designed to align with local procedures including concussion determination and RTP decisions.^19,20^ Research coordinators attended games and were alerted to players with a concussion. An attempt was made to record from concussed athletes early, and all recordings except 1 were conducted within 1 hour of the index concussive event. Coordinators traveled to individuals’ homes every 1 to 3 days for a month after injury to obtain additional recordings. The A2 cohort was invited to wear a wristband accelerometer after injury to document physical activity (Alphabet).

#### Headpulse Recordings

The headpulse comprises forces aimed toward the head following each cardiac contraction in the 10 to 15 milli-g (g, gravitational force unit) range. A noninvasive battery-powered device of highly sensitive accelerometers attached to a headband, placed on participants’ heads coronally, recorded headpulse (eFigure 1 in Supplement 1). In A1, bilateral triaxial accelerometers were placed anterior to the ear over the temporal bone. In A2, a second generation commercial device (MindRhythm, Inc) with a unilateral triaxial accelerometer was used. Electrocardiogram (ECG) was transduced using a standard 3-lead system to provide heart contraction timing and heart rate. Devices connected to a smartphone with Bluetooth and custom software (Apple). Recordings lasted 180 seconds in A1, 90 seconds in A2, and were obtained in the seated position. Participants were asked to hold their head still and not speak or chew. Gross body motion overpowers headpulse transduction and in A1 excessive body movement degraded several recordings. In A2, the second-generation device provided feedback to participants when motion was detected leading to fewer excluded recordings. Participants completed an adapted digital Neurobehavioral Symptom Inventory (NSI)^22,23^ (eFigure 2 in Supplement 1) on the smartphone with each recording. Data were offloaded via micro-Secure Digital card. Athletes and study personnel were masked to analyses, and headpulse did not factor into RTP protocols.

#### Activity Tracking

Some A2 participants consented to wristband accelerometry recordings (Alphabet). Fairly active and very active number of minutes provided physical activity information.

### Statistical Analysis

#### Data Analysis and Biometric Abnormality

Data files were submitted to custom software (MATLAB version 2022a [MathWorks]) to analyze frequency data as previously described.^10^ Time domain accelerometry signals from the right-sided accelerometer for 45 heart beats were converted to frequency domain using Fourier transformation. The average heart rate during recordings was derived from R-wave analysis of the ECG, providing heart rate fundamental and harmonics. The frequency transform was sampled at the fundamental and successive harmonics 2 through 9. Factors R1 and R2 were calculated as the ratio of the mean of the fifth and sixth harmonics divided by the maximum of the harmonics 1 through 3 (R1), and the mean of the seventh and eighth harmonic divided by the maximum of harmonics 1 through 3 (R2) (eFigure 3 in Supplement 1).^10^ The mean and standard deviations of R1 and R2 values for controls were calculated. To compare individual participant data with controls, sex-specific mean R1 and R2 values were subtracted from concussed individual R1 and R2 values for each recording, then divided by the control mean standard deviation, providing a Z score. Higher *Z* scores represent higher frequency shift of headpulse following injury.^4,10,11^ We defined any recording with *Z* greater than 2 as significant, and the earliest value to exceed this threshold was defined as biometric (or headpulse) onset of abnormality. The threshold for significance was *P* < .025.

#### Symptomatic Duration

We defined time between concussion determination (day of injury) until resolution of individual NSI score to zero as symptomatic duration. *Z* scores and contemporaneously acquired NSI scores were plotted over time. If an individual did not return to and stay at an NSI of zero during their recording period, they were considered persistently symptomatic.

#### Biometric Abnormality Duration

For individuals with biometric onset, the biometric offset was defined as time that *Z* scores fell and stayed below 1 through end of recording period. The choice of 1 SD was based on the observation that *Z* scores fell over time in a characteristic fashion, and allows for objective quantization of biometric-defined concussion parameters. Kaplan-Meier curves were generated for symptomatic duration and biometric abnormality duration.

## Results

**Figure 1.**
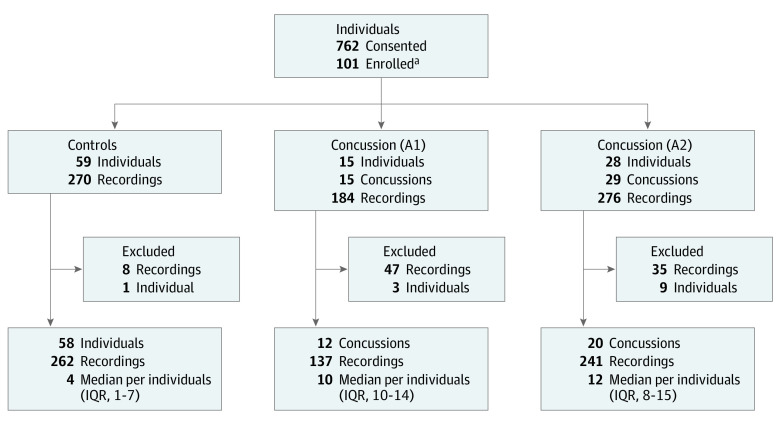
Study Flow Chart Study cohorts drawn from the Australian and University of California San Francisco Concussion Study in Athletes (AUSSIE), which included feasibility (A1) and validation (A2) phases. An individual was considered enrolled if they provided at least 1 headpulse recording. One concussed individual in the A2 cohort sustained 2 concussions separated by more than 1 month. Recordings with excess body motion or poor quality electrocardiogram recordings were excluded; this led to the exclusion of 1 control participant. Biometric onset and offset analysis required 2 or more recordings in the first week following injury; 12 individuals were excluded (5 because of poor quality recording and 7 withdrew after the first recording) resulting in 32 individuals for temporal analysis. ^a^One participant who started as a control was later concussed and was counted in control and concussion cohorts (so the sum of control and concussed cohorts was 102 individuals).

Of 762 consented athletes, 59 control and 43 concussed individuals had headpulse measurements (Figure 1). A control individual was later concussed (counted once in the control cohort and once in the participant cohort, reflected in numbers above) and a concussed individual in A2 sustained a second concussion (separated by more than 1 month), yielding 44 total concussions from 101 total individuals. A1 (all male) included 17 control (median [IQR] age, 23 [19-28] years) and 15 concussed individuals (median [IQR] age, 21 [19-23] years) (Table). A2 included 25 female (median [IQR] age, 21 [20-22] years) and 17 male (median [IQR] age, 26 [23-29] years) control individuals, and 8 female (median [IQR] age, 28 [20-31] years) and 20 male (median [IQR] age, 21 [19-23] years) concussed individuals. Cohorts had similar years of education. Female athletes had fewer self-reported prior concussions than males. Male athletes had played more games than female athletes but there were no significant sex differences in games played between control and concussion participants.

**Table.** Participant Demographic Information

Characteristics	Median (IQR)			
	AUSSIE-1 control	AUSSIE-1 concussion	AUSSIE-2 control	AUSSIE-2 concussion
Sex, No. (%)				
Female	0	0	25 (60)	8 (29)
Male	17 (100)	15 (100)	17 (40)	20 (71)
Age, female, y	NA	NA	21 (19.5-21.5)	27.5 (19.8-31.3)
Age, male, y	23 (19-27.5)	21 (19-22.8)	26 (22.5-28.5)	21 (19-22.8)
Education, female, y	NA	NA	14 (12-15)	16 (12.3-17)
Education, male, y	14 (12-15)	15 (12-15)	17 (13.5-17.5)	12 (12-15)
Prior concussions, female (%)	NA	NA	8 (32.0)	3 (37.5)
No. concussions, Female	NA	NA	1 (1-0)	2 (1-10)
Latency of last concussion, female, y	NA	NA	5 (2-11)	5
Prior concussions, male (%)	11 (64.7)	11 (73.3)	7 (41.2)	14 (70.0)
Prior concussions, male	2 (1-4)	2 (2-4)	2 (1-3)	2.5 (2-4)
Latency of last concussion, male, y	3.5 (1.9-7.5)	3.5 (2.6-5.3)	6.5 (4-10)	3 (1.5-6)

Abbreviations: AUSSIE, Australian and University of California San Francisco Concussion Study in Athletes; NA, not applicable.

### Diagnosis of Concussions

Real-time concussive events were identified by team staff or by symptomatic players who then exited field of play for assessment. Events were identified by a physician in 7 cases (17%), other team staff member in 25 (61%), the player in 8 (20%), and in 1 case by an unknown method (2%). Players were evaluated and diagnosed with concussions per Australian Football League guidelines^19,20^ before headpulse collection, and 4 (10%) had loss of consciousness (LOC), 16 (39%) had alteration in consciousness, and 7 (17%) had transient posttraumatic amnesia (PTA). No participant sustained a concussion within the month following injury.

### Headpulse Recording Yield

In the feasibility phase, 40 of 184 recordings (22%) were motion degraded. In A2, we used an improved device that provided feedback regarding excessive body motion resulting in fewer rejected recordings (24 of 276 recordings [9%]).

### Control Headpulse

**Figure 2.**
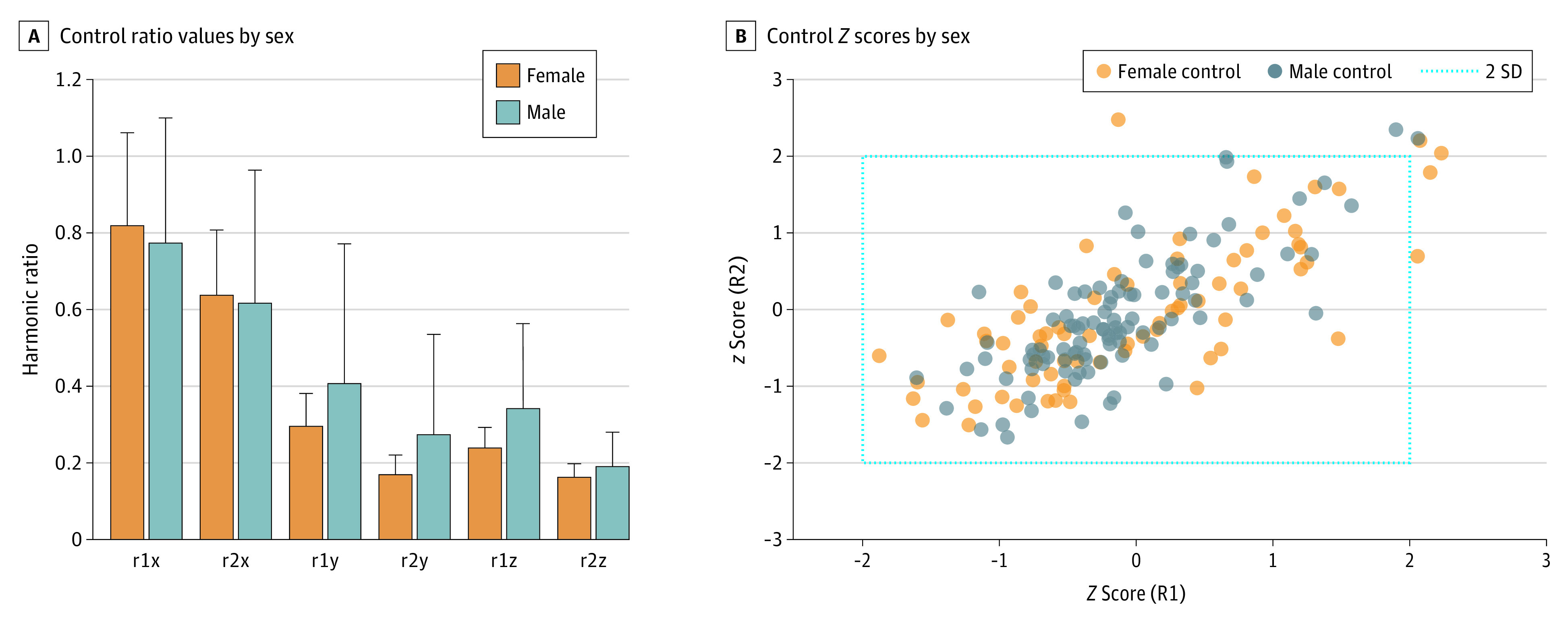
Control R1 and R2 Value *Z* Scores A, R1 and R2 values for cranial accelerometer vertical (x), anterior-posterior or AP (y), and right-left or lateral (z) axes, where x-axis represents accelerometer axis designation, and the y-axis represents absolute R1 and R2 ratio values (for formulae, see Methods; eFigure 3 in Supplement 1). Error bars indicate 1 SD. There were no significant sex differences for R1 or R2 values. B, *Z* score plot for each recording, showing majority of recordings within 2 standard deviations of mean values.

Male athletes in A1 and male and female athletes in A2 provided control data, and A2 controls provided more recordings per individual compared with A1. R1 and R2 ratios were calculated for each recording. If an individual provided multiple recordings, then R1 and R2 means were calculated. R1 and R2 means (if multiple recording) plus single recording values were combined and averaged and standard deviations were generated for each accelerometer axis (Figure 2). There were no significant sex differences between R1 and R2 values within axes. The average and standard deviation values were used to analyze *Z* score data from concussed participants.

### Participant Headpulse

Two illustrative examples of concussed participants are provided in eFigure 4 in Supplement 1. To explore headpulse sensitivity for predefined biometric abnormality onset and offset thresholds, concussed individuals with more than 2 noise-free recordings during the first week were examined; 7 declined participation after first recording and 5 had degraded recordings, leaving 32 concussions for analysis (12 from A1, 20 from A2). In A1, 10 of 12 concussions (83%) met biometric onset threshold within first 7 days. In A1 and A2 combined, 26 of 32 concussed athletes (81%) met the threshold within the first 7 days. Overall, headpulse analysis detected 9% of concussions on day 0, 50% by day 2, and 90% by day 14. Since LOC and PTA during index event may represent more severe concussive injury,^24^ we specifically reviewed individuals with LOC, PTA, or both to query pattern aberrations. No differences in maximum biometric value or recovery course were seen for those with LOC, PTA, or both compared with those without either sign or symptom.

**Figure 3.**
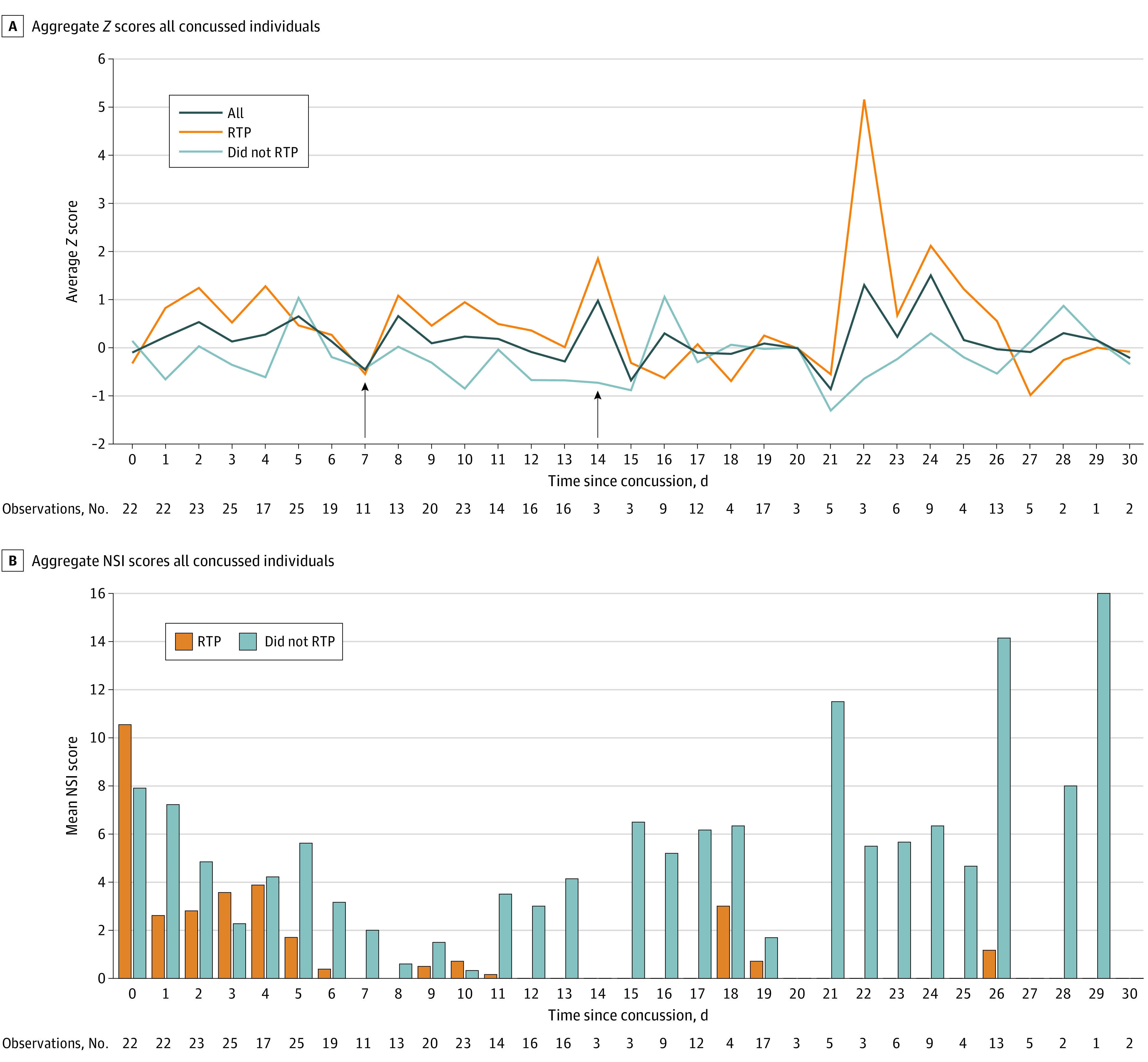
Biometric *Z* Scores and Neurobehavioral Symptom Inventory (NSI) Scores for 30 Days Following Concussion A, individuals who did return to play (RTP) had marked increases in *Z* scores in the latter half of the month compared with those who did not. Most players who RTP had done so by day 14 (5 by day 7, 11 by day 14; indicated with black arrows). B, NSI scores plotted for individuals who RTP compared with those who did not showed that most individuals who RTP were symptom free for the second half of the month. NSI scores were lower in those who RTP.

Averaging across days reduced the aggregate *Z* score because individuals had differing days of concussion onset (Figure 3). The largest discrepancy in daily averaged *Z* scores occurred in individuals with RTP in second half of the month. Those with RTP were mostly asymptomatic.

### Symptomatic Duration

**Figure 4.**
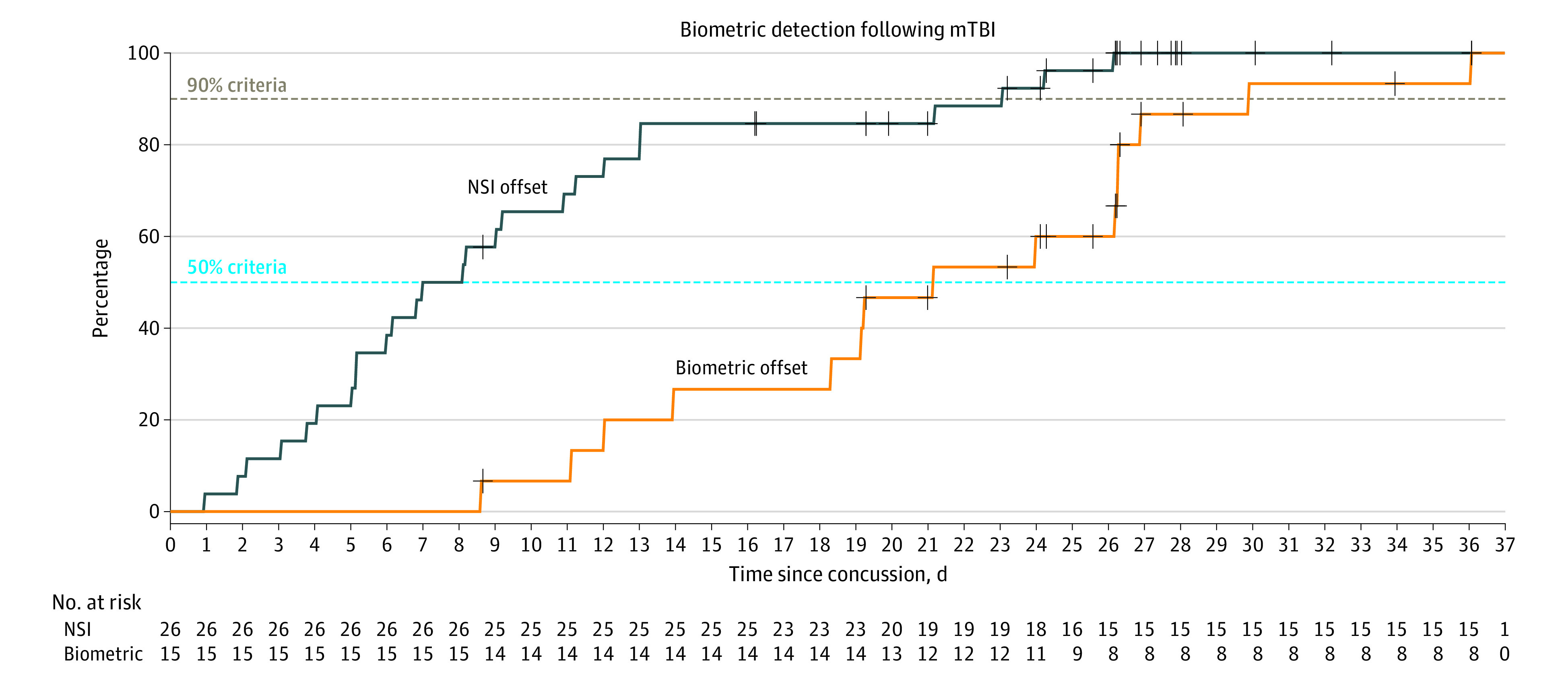
Time Course of Symptom Resolution Compared With Biometric Offset Time course of symptom resolution compared with biometric offset in the A1 and A2 cohorts who had more than 1 recording (32 individuals). Comparison of concussion recovery by Neurobehavioral Symptom Inventory (NSI) returning to zero and return of biometric ratios below 1 SD from the mean of normal. By 30 days, 26 of 32 participants (81%) had returned to a zero NSI score compared with 15 of 26 participants (58%) who had biometric onset then returned to normal biometric values. Curves are normalized to the number of individuals that had each biometric offset by end of 30-day period; each biometric ends at 100% to allow comparisons in timing. Black plus marks indicate time of censoring (time beyond which a participant had no further data). By day 7, 50% of participants were asymptomatic, yet by day 19, 50% of participants had biometric offset, suggesting that biometric abnormalities resolve around 14 days later.

Of 32 participants with longitudinal recordings, 26 had NSI values returning to zero within 30 days. The overall symptomatic duration is shown in Figure 4. Of the 81% of athletes that had symptomatic duration under 1 month, 50% returned to a zero NSI score by day 7.

### Biometric Abnormality Duration

For biometric abnormality duration, we considered individuals with at least 1 recording with Z score above 2 that returned to under 1 at or before last recording (15 individuals). Compared with symptom resolution, only 57% of athletes demonstrated biometric resolution by day 30 with 50% achieving this by day 21, 14 days later than NSI improvement (Figure 4).

### Activity Tracking

eFigure 5 in Supplement 1 shows an example of the association between activity and headpulse biometric. Six individuals wore the activity tracking device in A2, and 4 of 6 used the device for more than 1 day. All 4 had later increases in *Z* scores above 2 after tracking device–determined physical activity 2 to 8 days following concussion. An exercising control participant volunteered to use the device for 1 month and provided 16 recordings to demonstrate association between recent physical activity and biometric measures (eFigure 6 in Supplement 1).

## Discussion

To our knowledge, this is the largest series of longitudinal headpulse measurements following concussion, expanding on prior findings.^10^ We demonstrated feasibility of data collection and analysis (A1) then validated findings in A2, which additionally included female athletes. While controls were evaluated in Auerbach et al^25^ (82 participants) and a recent case series (5 participants), female athletes were not included. Headpulse *Z* scores met our prespecified threshold for postconcussive biometric onset but there was a 1-3–day lag after injury. Such changes were not observed in actively exercising control individuals.

Biometric abnormality onset was not always concordant with symptom endorsement. After RTP (15 individuals) or other activities (inferred from 4 device-monitored participants) following concussion, *Z* scores rose or remained elevated despite low or no symptoms. Auerbach et al^10^ included 13 concussed and 82 normal control high school US football athletes; case examination revealed delayed headpulse abnormality designation with gradual return to normal patterns, but again after symptom resolution. We used the same algorithm, replicating these delays in a larger population with improved injury detection sensitivity of 81% compared with prior methods (77%).^10^ We demonstrated group and individual differences between athletes with SRC and controls particularly after RTP. Most daily aggregate headpulse values stratified by day after concussion did not meet our biometric abnormality onset threshold in part due to combining results from differing days following SRC (Figure 3). However, examination of individual-level concussion headpulse patterns more clearly demonstrated headpulse abnormality onset and offset (eFigures 4, 5 in Supplement 1), and examining biometric and symptom duration onset and offset over time demonstrated a 14-day lag between symptom resolution and biometric normalization (Figure 4).

For the few participants with activity device tracking–quantification, etiology of *Z* score elevations following unstructured activity is not yet known. Care must be taken to interpret this and other RTP biometric rises given demonstrated efficacy of structured exercise as a treatment for concussion in sports and military settings.^26,27,28,29,30,31,32^ A potentially relevant finding in our study is the persistence of symptoms for those with delayed RTP or with low or no activity levels (Figure 3); it is not clear if this is attributed to RTP protocol adherence due to persisting symptoms,^19,20^ or persisting symptoms due to lack of activity. The association between headpulse and activity, including exercise, requires further study.

The current concussion biomarker landscape includes candidate metrics of differing modalities. Among blood-based metrics, GFAP and UCH-L1 are elevated in SRC and may have prognostic value.^24^ McCrea and colleagues^24^ found that GFAP and UCH-L1 levels were highest acutely while neurofilament light chain levels increased over days in those with LOC and PTA, and we did not note any additional biometric aberrations in our participants with either or both LOC and PTA. Candidate neuroimaging metrics are not yet validated for routine clinical practice and include diffusion tensor imaging, resting or task-based functional imaging, and markers of cerebrovascular reactivity.^33^ Both acute and long-term changes have been noted across a variety of these imaging metrics,^6,33,34^ as with blood-based tests. Also of interest are individual genetic and inflammatory profiles, which may affect symptom burden or duration,^6^ along with factors such as previous concussion or comorbid conditions. The delay in headpulse abnormalities, with further rise of *Z* scores following RTP or early unstructured activity, aligns with other metrics that demonstrate time dependence.

The etiology of headpulse harmonic shifts to higher *Z* scores is not yet known. We also cannot determine directionality of changes with respect to injury, compensatory response to injury, or recovery. Concussion triggers overlapping, interrelated events including cell membrane–associated ionic shifts, neurotransmitter release, cerebral blood flow and vascular reactivity changes, and metabolic crisis.^35^ Speculative causes of SRC headpulse signal changes therefore include alterations in brain parenchymal mechanical resonance (ie, stiffer brain) induced by concussive injury, modulated by vascular response. Heart rate harmonics are central to headpulse derivation, and SRC-related autonomic dysfunction may contribute to headpulse changes. Renewed interest in ballistocardiography has produced improved signal analysis and remote monitoring techniques,^36,37,38^ and findings within cardiovascular fields^8,9,37,39^ may offer etiological and signal analysis insights. Blood pressure and heart rate likely affect headpulse and require further study.

Headpulse patterns changed in a characteristic manner in SRC. Like some candidate biomarkers,^40,41^ headpulse remains altered beyond symptom resolution. Headpulse changes may also be associated with activity, and this warrants further investigation, along with cardiovascular metrics, to determine directionality. Sports-related concussion and recovery remain clinical determinations. Objective biomarkers will ultimately support personalized RTP protocols, minimizing risk of subsequent concussive events while preserving the spirit and benefits of sport. Next steps include determining feasibility of headpulse device self-administration by concussed individuals, broader activity tracking to understand the headpulse-activity relationship, and enhanced headpulse characterization via cognitive and clinical assessments.^4^

### Strengths and Limitations

Our study has several strengths. Participants included female athletes, who are understudied in SRC. Longitudinal recordings identified the trajectory of headpulse pattern changes. The NSI was collected with each headpulse recording, revealing the mismatch between headpulse and reported symptoms. Participants and research coordinators were masked to analyses. Headpulse analysis did not affect RTP decisions; this allowed for generalizability in amateur or recreational settings given observation of some individuals’ RTP before the required 12-day abstinence period in Australian Rules Football.^19,42^

This study had several limitations. Study points of entry and exit were not standardized with preseason or postseason headpulse testing; however, no baseline recording was necessary given established control biometric data. Longitudinal headpulse recording schedules were not strictly standardized across time for participants. Seven concussed individuals provided 1 recording before withdrawing, and 72 of 723 recordings were motion degraded but this prompted procedure and device improvements in A2. Concussion history was elicited by self-report (which identified a high prevalence of prior concussion). It is also possible that some concussions were not recognized by team staff or athletes during play. Despite these limitations, cranial accelerometry offers a simple, noninvasive, easy to use method for headpulse determination.

## Conclusions

In this study, we found that headpulse patterns characteristically changed after SRC and remained altered beyond symptom resolution. Headpulse pattern associations with activity and with cardiovascular metrics warrant further investigation to refine directionality.

## Notes

Supplement 1. **eFigure 1.** Study Devices **eFigure 2.** Digitized NSI **eFigure 3.** Headpulse Harmonics **eFigure 4.** Subject-Level Headpulse Examples **eFigure 5.** Biometric and Activity Levels **eFigure 6.** Male Control Subject With Longitudinal Headpulse and Activity Tracking

Supplement 2.
Data Sharing Statement
