# Supplementary material for: Headpulse Biometric Measures Following Concussion in Young Adult Athletes
Source: JAMA Netw Open. 2023 Aug 11;6(8):e2328633. doi: 10.1001/jamanetworkopen.2023.28633 (PMC10422194; doi:10.1001/jamanetworkopen.2023.28633)
Supplement: Supplement 1. — eFigure 1. Study Devices eFigure 2. Digitized NSI eFigure 3. Headpulse Harmonics eFigure 4. Subject-Level Headpulse Examples eFigure 5. Biometric and Activity Levels eFigure 6. Male Control Subject With Longitudinal Headpulse and Activity Tracking [file jamanetwopen-e2328633-s001.pdf]

Supplemental Online Content

Halabi C, Norton L, Norton K, Smith WS. Headpulse biometric measures following concussion in young adult athletes. *JAMA Netw Open.* 2023;6(8):e2328633. doi:10.1001/jamanetworkopen.2023.28633

eFigure 1. Study Devices

eFigure 2. Digitized NSI

eFigure 3. Headpulse Harmonics

eFigure 4. Subject-level Headpulse Examples

eFigure 5. Biometric and Activity Levels

eFigure 6. Male Control Subject With Longitudinal Headpulse and Activity Tracking

This supplemental material has been provided by the authors to give readers additional information about their work.

© 2023 Halabi C et al. *JAMA Network Open.*

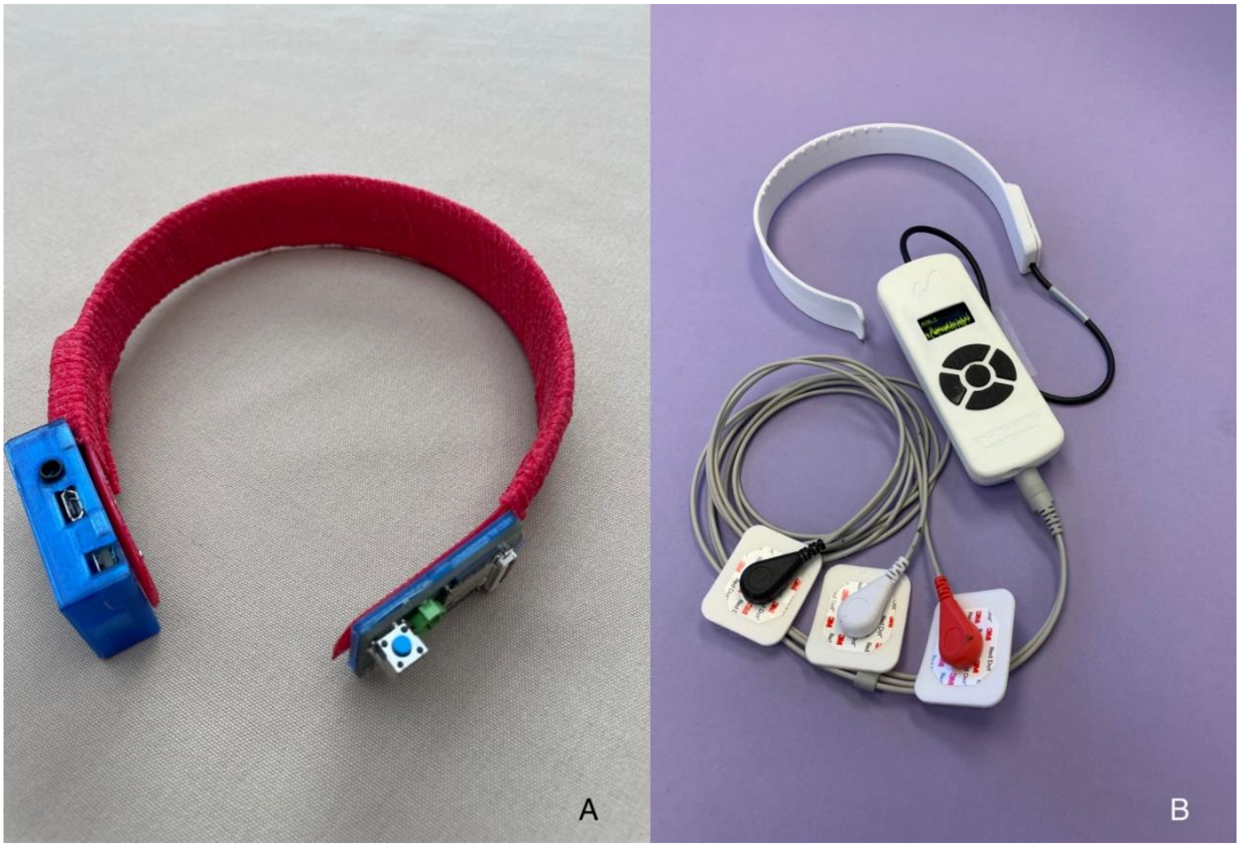

eFigure 1. **(A) Headset used in A1 cohort, designed at UCSF.** Two circuit boards were mounted on a plastic hairband and interconnected by a ribbon cable and wrapped in tape. Protective cap removed for illustration. Circuit boards each contain a sensitive 3-axis accelerometer that is sampled at 250 samples/sec and stored on a micro-SD card. The device has Bluetooth interface to the custom iPhone app shown in Figure e2. ECG leads are attached via cable that plugs into the headset (not shown). **(B) Commercial headset used in A2 cohort.** The accelerometer is identical to the device in (A) and interfaces to the custom iPhone App.

© 2023 Halabi C et al. *JAMA Network Open.*

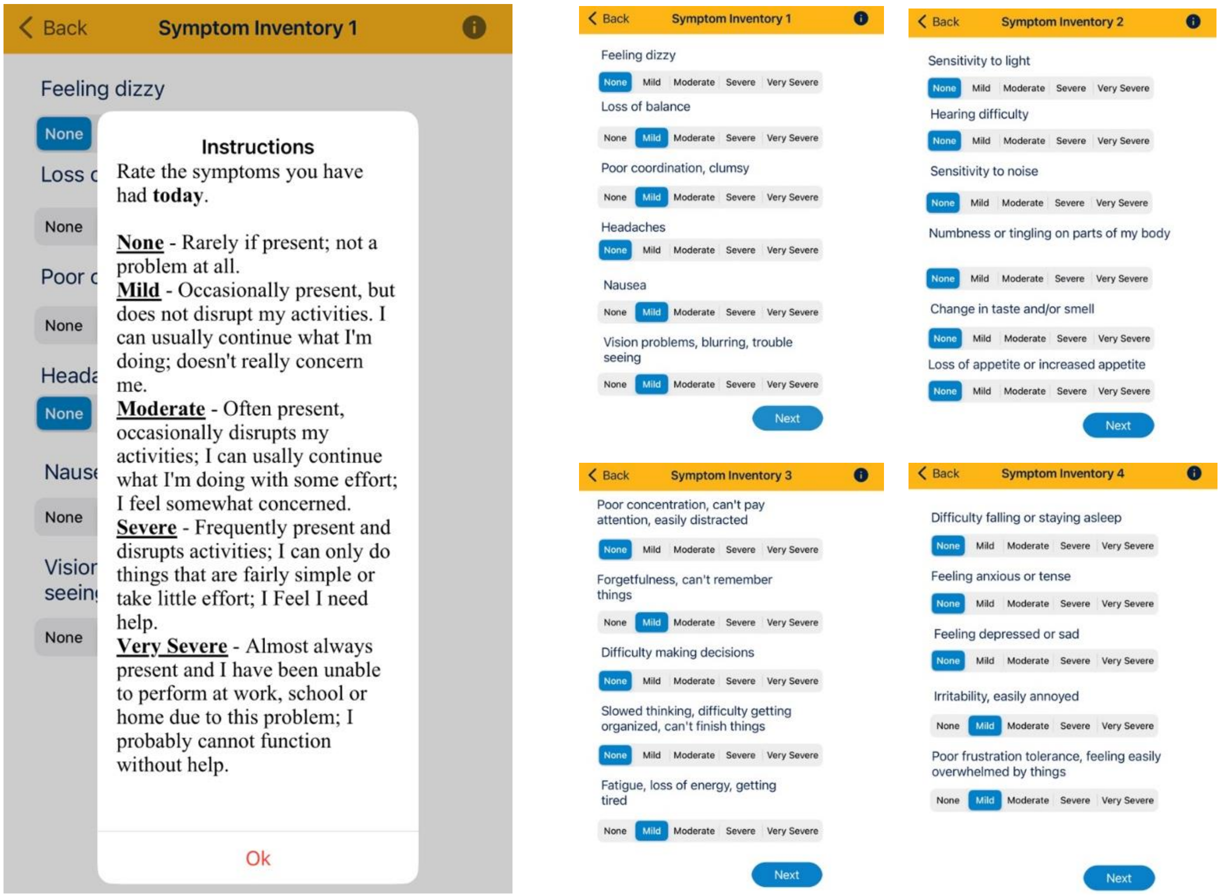

eFigure 2. iPhone screenshots of the digitized Neurobehavioral Symptom Inventory (NSI).

Subjects completed these questions for each recording.[1]

© 2023 Halabi C et al. *JAMA Network Open.*

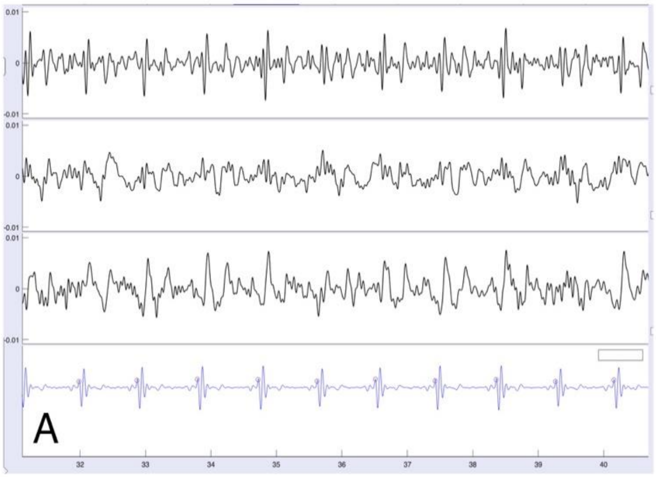

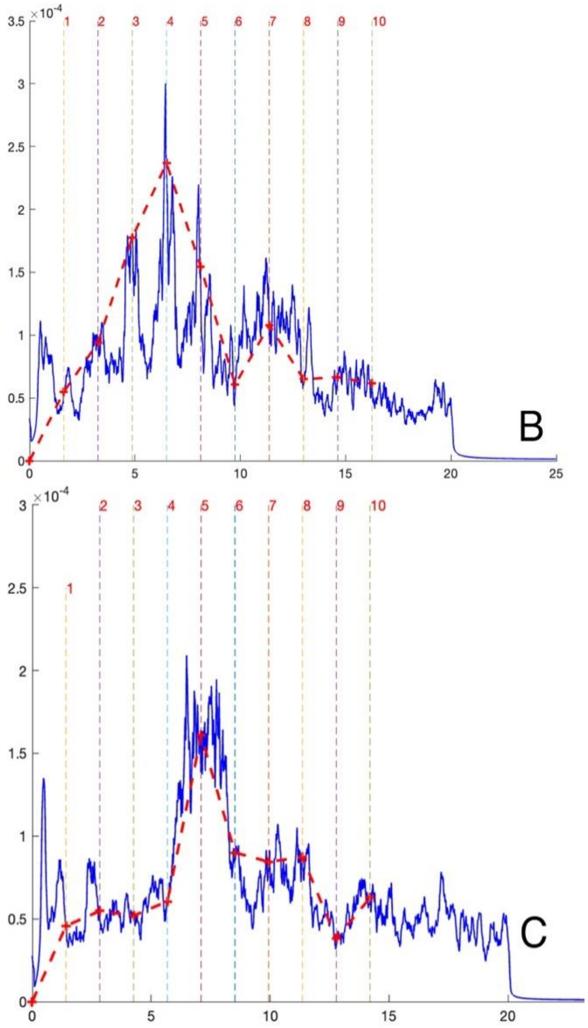

eFigure 3. The headpulse. (A) Typical headpulse recording. Top three panels are headpulse forces (in g) in vertical, anterior-posterior, and lateral axes, respectively. Bottom trace is ECG. X-axis is time (sec). **(B)** Frequency domain of a subject immediately after concussion. Harmonics of the heart rate are shown as vertical dotted lines. The height of the frequency transform at each harmonic is used to calculate R1 and R2. Harmonics 5 and 6 are smaller than the maximum of harmonics 1-3. **(C)** Same subject 3 days later showing that harmonics 5 and 6 are now greater than the maximum of harmonic 1-3, indicating concussion. X-axis is frequency in Hz.

© 2023 Halabi C et al. *JAMA Network Open.*

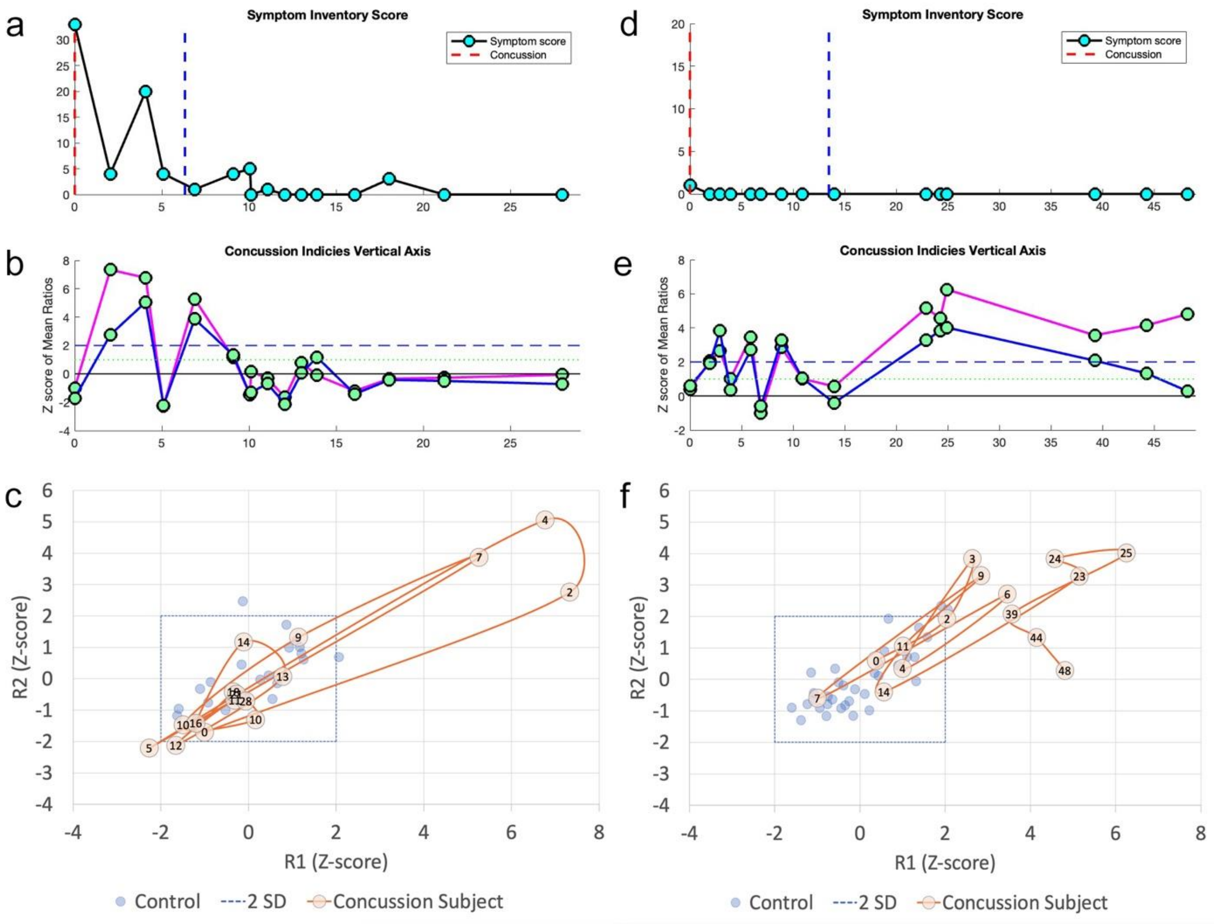

Figure e4. Subject-level headpulse examples. (A-C) Female subject who sustained a concussion during an Australian Football game; SRC characteristics included head strike and ataxia of gait. **(A)** NSI score time locked to each headpulse recording over the course of a month. The vertical dashed orange line is the time of concussion and the blue line represents RTP. **(B)** 16 headpulse recordings obtained while resting in seated position. R1 (magenta) and R2 (blue) values are shown for each recording. Ordinate values are Z-scores of R1 and R2 values compared to the mean of controls; a dashed line at 2 indicates 2-standard deviations above control means, and another at 1 indicates 1 SD. The first recording crossing the 2-SD line in any axis is defined as biometric onset time of concussion. As was typical of both cohorts, the Z scores exceed 2 on day 2, return to normal on day 5-7 then rise and fall again in an 'M-shaped' fashion. This subject was sedentary until she returned to play. This is an example where the biometric signal parallels the NSI. X-axis in **(A,B)** is days since concussion. **(C)** Plot of R1 and R2 Z scores for all female controls (blue markers) and the subject (orange). The number of days

© 2023 Halabi C et al. *JAMA Network Open.*

following concussion is indicated inside the markers. Points falling out of the dotted box are considered abnormal. **(E-F)** 21-year-old male was knocked to the ground, stood up and was ataxic then fell. The layout is like panels a-c and subject provided 15 recordings. Despite having symptoms the first day and none thereafter, this subject's biometric later rose above 2 SD three times, returned to normal (below 1 SD) then rose again for the remainder of the month following RTP. This is an example where the biometric signal is independent of the NSI, i.e. the concussed athlete is asymptomatic but headpulse is altered. X-axis in (D,E) is days since concussion.

© 2023 Halabi C et al. *JAMA Network Open.*

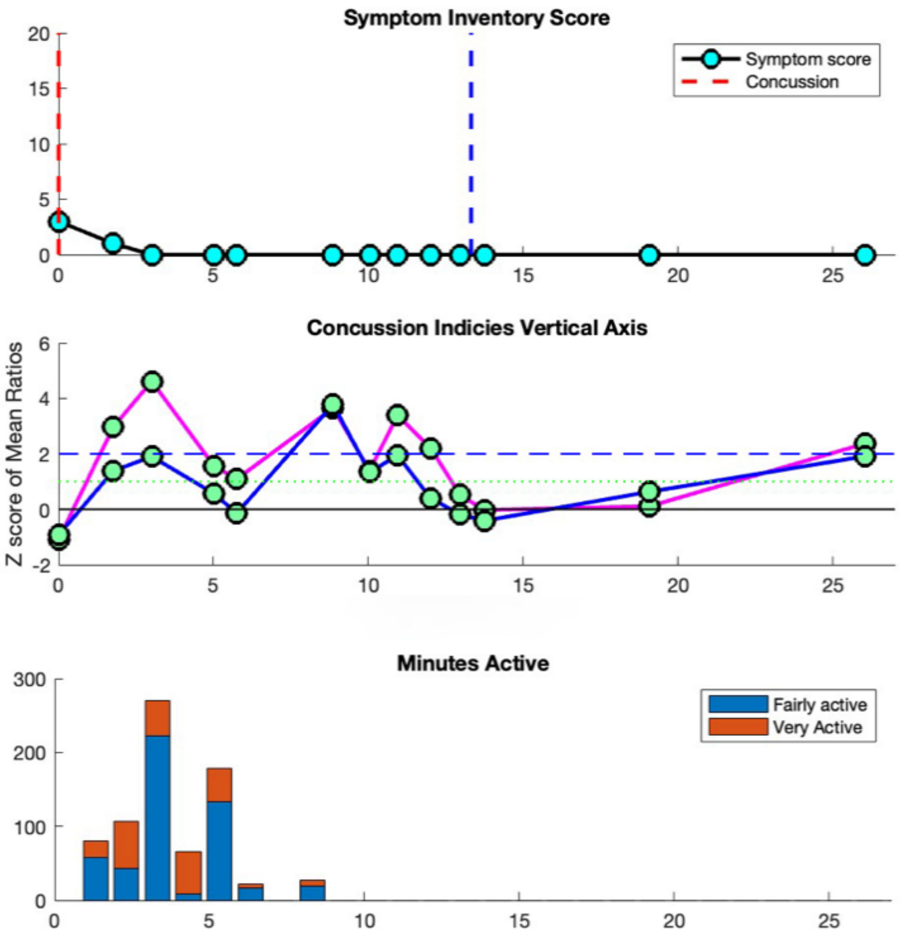

Figure e5. Biometric and activity levels. A 19-year-old male subject with no known prior concussion sustained a concussion during an Australian Football game. X-axis is days since concussion. Y-axis is NSI score (top row), biometric R1/R2 Z scores from vertical accelerometer axis (middle row), and active minutes (bottom row). In addition to headpulse recordings, the subject wore a wrist-mounted accelerometer. Activity is defined as 'fairly active' and 'very active' by the device manufacturer (FitBit, Alphabet, San Francisco, CA). This device documented physical activity before RTP on day 14. The subject discontinued the exercise tracking device on day 14. Headpulse met biometric onset threshold coincident with activity levels despite low or no symptoms.

© 2023 Halabi C et al. *JAMA Network Open.*

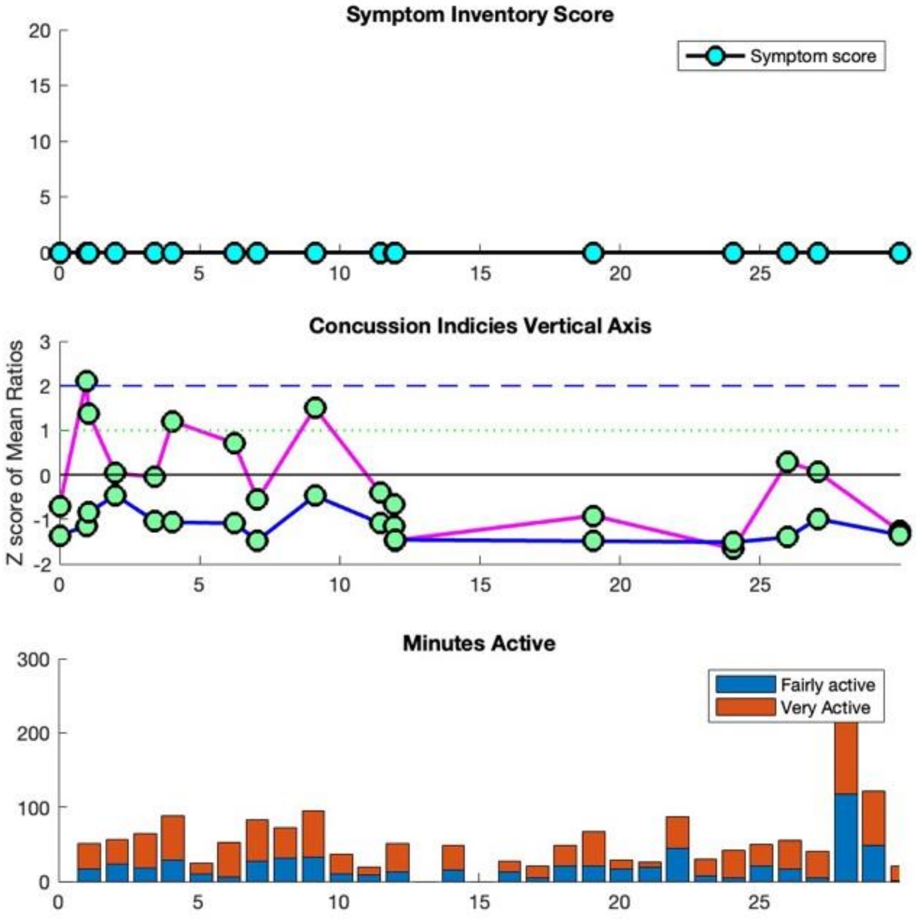

Figure e6. **Male control subject with longitudinal headpulse and activity tracking**. Subject provided 16 headpulse recordings during a month and wore a wrist accelerometer. He did not endorse symptoms on NSI and did not sustain a concussion during the period of observation. The second performed recording slightly exceeded 2 SD above control R1 values as is expected by chance (16 recordings each for R1 and R2, 2.5% (single tail) expected to exceed the threshold, or 0.975 ^ 32 = 0.44). However, the trajectory of his Z scores do not resemble what is seen in concussion cases (see Figure e4).

© 2023 Halabi C et al. *JAMA Network Open.*

References

1. Cicerone KD, Kalmar K. Persistent postconcussion syndrome. *Journal of Head Trauma Rehabilitation*. 1995/06// 1995;10(3):1-17. doi:10.1097/00001199-199510030-00002

© 2023 Halabi C et al. *JAMA Network Open.*
