# Supplementary material for: Headpulse Biometric Measures Following Concussion in Young Adult Athletes
Source: JAMA Netw Open. 2023 Aug 11;6(8):e2328633. doi: 10.1001/jamanetworkopen.2023.28633 (PMC10422194; doi:10.1001/jamanetworkopen.2023.28633)
Supplement: Supplement 2. — Data Sharing Statement [file jamanetwopen-e2328633-s002.pdf]

Data Sharing Statement

Halabi. Headpulse Biometric Measures Following Concussion in Young Adult Athletes. *JAMA Netw Open*. Published August 11, 2023. doi:10.1001/jamanetworkopen.2023.28633

Data
Data available: Yes
Data types: Deidentified participant data, Data dictionary
How to access data: Request for data can be sent to Wade.Smith@ucsf.edu
When available: beginning date: 07-01-2024

Supporting Documents
Document types: None

Additional Information
Who can access the data: Researchers whose proposed use of the data has been approved.
Types of analyses: For specified purpose after review.
Mechanisms of data availability: After approval of proposal and signed data access agreement.
